# Analysis of the Organic Hydroperoxide Response of *Chromobacterium violaceum* Reveals That OhrR Is a Cys-Based Redox Sensor Regulated by Thioredoxin

**DOI:** 10.1371/journal.pone.0047090

**Published:** 2012-10-11

**Authors:** José F. da Silva Neto, Caroline C. Negretto, Luis E. S. Netto

**Affiliations:** Departamento de Genética e Biologia Evolutiva, Instituto de Biociências, Universidade de São Paulo, São Paulo, Brazil; Wayne State University, United States of America

## Abstract

Organic hydroperoxides are oxidants generated during bacterial-host interactions. Here, we demonstrate that the peroxidase OhrA and its negative regulator OhrR comprise a major pathway for sensing and detoxifying organic hydroperoxides in the opportunistic pathogen *Chromobacterium violaceum*. Initially, we found that an *ohrA* mutant was hypersensitive to organic hydroperoxides and that it displayed a low efficiency for decomposing these molecules. Expression of *ohrA* and *ohrR* was specifically induced by organic hydroperoxides. These genes were expressed as monocistronic transcripts and also as a bicistronic *ohrR-ohrA* mRNA, generating the abundantly detected *ohrA* mRNA and the barely detected *ohrR* transcript. The bicistronic transcript appears to be processed. OhrR repressed both the *ohrA* and *ohrR* genes by binding directly to inverted repeat sequences within their promoters in a redox-dependent manner. Site-directed mutagenesis of each of the four OhrR cysteine residues indicated that the conserved Cys21 is critical to organic hydroperoxide sensing, whereas Cys126 is required for disulfide bond formation. Taken together, these phenotypic, genetic and biochemical data indicate that the response of *C. violaceum* to organic hydroperoxides is mediated by OhrA and OhrR. Finally, we demonstrated that oxidized OhrR, inactivated by intermolecular disulfide bond formation, is specifically regenerated via thiol-disulfide exchange by thioredoxin (but not other thiol reducing agents such as glutaredoxin, glutathione and lipoamide), providing a physiological reducing system for this thiol-based redox switch.

## Introduction

Reactive oxygen species (ROS), such as superoxide, hydrogen peroxide (H_2_O_2_) and organic hydroperoxides, are generated as by-products of aerobic metabolism and are also important components of plant and animal innate immune systems. Increased levels of these oxidants can damage DNA, proteins and membranes [1]. Lipid peroxidation, for instance, can occur due to the oxidation of polyunsaturated fatty acids by free radicals present on cell membranes or catalyzed by lipoxygenases, enzymes produced as part of the active defense response of plants and animals [2]–[5]. In addition to affecting membrane fluidity, lipid peroxides are highly toxic because their degradation results in the generation of more reactive by-products, such as acrolein and malondialdehyde (MDA). These short-chain aldehydes are capable of forming adducts with proteins and DNA, thus affecting essential cellular processes [6], [7]. Therefore, organic hydroperoxide detoxification is critical for bacterial survival.

The first characterized system for the detoxification of organic hydroperoxides in bacteria was the enzyme alkyl hydroperoxide reductase AhpCF [8], which is composed of two subunits, the thiol-dependent peroxidase AhpC (a member of the peroxiredoxin family), and AhpF, a flavoenzyme that reduces AhpC, using NAD(P)H reducing equivalents [9]. The AhpCF system is also an important scavenging enzyme for H_2_O_2_ [10], and accordingly, it is often regulated by OxyR, the master hydrogen peroxide-stress regulator in *E. coli* [1], [11].

A second system for organic hydroperoxide scavenging is the Ohr (organic hydroperoxide resistance) protein, initially described in *Xanthomonas campestris* [12]. A series of genetic and expression studies in several bacteria indicate that the gene *ohr* is specifically induced by organic hydroperoxides. Furthermore, *ohr* mutants are hypersensitive to organic hydroperoxides, not being affected by other agents that generate ROS [12]–[16]. In fact, *in vitro* biochemical studies have demonstrated that Ohr is a Cys-based, thiol-dependent peroxidase able to reduce organic hydroperoxides more effectively than H_2_O_2_ [17], [18]. Ohr has a low similarity to OsmC (osmotically inducible protein), a member of the osmotic stress response in *E. coli* [19]. Despite the fact that both proteins are endowed with a thiol peroxidase activity and share a common structural fold [17], [20], [21], they display different patterns of regulation, probably reflecting diverse physiological roles. In fact, they are classified as two distinct subfamilies of the OsmC/Ohr family, and both are exclusively present in bacteria [22]. Recently, our group has shown that the peroxidase activities of both OsmC and Ohr are supported *in vivo* by lipoylated proteins, a previously undescribed enzymatic activity [23].

In many bacteria, the gene for Ohr is in a close vicinity to a gene for OhrR, a redox-sensing repressor of the MarR family. Members of this family act as dimeric proteins and control genes that confer resistance to multiple antibiotics and ROS [11]. In some bacteria, it has been shown that, in the reduced form, OhrR binds to the *ohr* promoter and, thereby, represses its transcription [14], [15], [16]. Upon oxidative stress stimulated by organic peroxides, OhrR is oxidized and suffers a conformational modification that decreases its affinity for the *ohr* promoter [11]. Indeed, structural data available for the OhrR proteins of *B. subtillis* [24] and *X. campestris* [25] indicate that the oxidation of cysteines leads to a rotation of the OhrR DNA-binding motif wHTH (winged helix-turn-helix) with subsequent DNA dissociation, resulting in the expression of the *ohr* gene. However, there are mechanistic differences among the OhrR proteins from different bacteria. In *Streptomyces coelicolor*, OhrR acts both as a repressor of *ohrA* as well as an activator of *ohrR* expression [15]. According to the mechanisms of redox regulation by organic hydroperoxides, OhrR proteins are classified into two subfamilies: the 1-Cys subfamily, which contains a single conserved cysteine that is best characterized in *B. subtilis*, and the 2-Cys subfamily, which contains the prototypical OhrR of *X. campestris* and contain two redox-active cysteine residues [11].

In *B. subtilis*, the oxidation of the highly conserved Cys15 residue of its 1-Cys OhrR produces a sulfenic acid intermediate [26], which then reacts with low molecular weight thiols, leading to the formation of mixed disulfides [27]. Alternatively, the sulfenic acid can suffer a condensation reaction with a primary amine, giving rise to a cyclic sulfenamide, or can be over-oxidized by other peroxide molecules to sulfinic or sulfonic acids [11], [27], [28]. Otherwise, the inactivation of the *X. campestris* 2-Cys OhrR repressor involves the formation of a reversible intermolecular disulfide bond between the two subunits of the OhrR dimer, involving the conserved Cys22 and Cys127 residues [11], [28], [29]. Although the oxidation of 1-Cys and 2-Cys OhrRs has been investigated in some bacteria, currently the identities of biological systems capable of reducing OhrR have not been described. Thiol-dependent regulators known to operate as authentic thiol-based regulatory switches include the hydrogen peroxide activator OxyR, which is deactivated by the glutaredoxin system [30] and the σ^R^-RsrA system, whose anti-sigma factor RsrA is reduced by the thioredoxin system [31] and by mycothiol [32].

Because Ohr/OhrR proteins are exclusively present in bacteria and present several unique features, they may represent promising targets for drug development. *Chromobacterium violaceum* is a Gram-negative β-proteobacterium widely distributed in the soil and water of tropical and subtropical areas worldwide, including diverse Brazilian ecosystems [33]. This saprophytic bacterium is also an opportunistic pathogen that infects both humans and animals. Human *C. violaceum* infections are rare but spread rapidly, leading to abscesses in lungs, liver and spleen and a high mortality rate, which is in part due to its resistance to a broad range of antibiotics [34], [35]. In addition to these clinical reports, it has been verified that mice with defective phagocyte NADPH oxidase, and thus unable to produce the oxidative burst, were more susceptible to infection by *C. violaceum* and *Burkholderia cepacia* [36]. More recently, the pathogenicity of *C. violaceum* has been studied in model organisms. It has been discovered that a quorum-sensing system contributes to *C. violaceum* virulence in *Caenorhabditis elegans* [37] and that the island 1 type III secretion system is a major virulence factor in the murine model [38].

In the environment or within the host organism, *C. violaceum* is expected to be exposed to oxidative stress conditions. Analysis of the *C. violaceum* genome sequence indicates the presence of many genes related to oxidative stress responses [39], [40]. However, the mechanisms employed by *C. violaceum* to resist oxidative insults, including organic hydroperoxide, are largely unknown. In this work, we show that OhrA and its negative regulator OhrR are critical for sensing and degradation of organic hydroperoxides in *C. violaceum*. Indeed, reduced OhrR bound to operator sites on the *ohrA* and *ohrR* promoters. Upon oxidation of a conserved cysteine residue, OhrR was functionally inactivated by intermolecular disulfide bond formation, resulting in a strong derepression of *ohrA* transcription. Remarkably, oxidized OhrR was specifically re-reduced *in vitro* by the thioredoxin system, among other thiol systems, restoring its DNA-binding activity. These results provide a reversible thiol-based switch mechanism to regulate the response of bacteria to organic hydroperoxide insults.

## Results

### Identification of OhrA and OhrR orthologues in *C. violaceum*

To obtain a complete catalog of Ohr proteins in the *C. violaceum* genome, we performed a search for the OsmC/Ohr family (PF02566) in the Pfam protein database, a comprehensive compendium of protein families [41]. The OsmC/Ohr family is widely distributed among bacteria (5039 protein sequences in 1916 species, according to Pfam version 26.0 November 2011) and contains proteins initially described as belonging to the OsmC and Ohr subfamilies [22] in addition to a third group of uncharacterized proteins represented by YhfA of *E. coli* [42]. Our search identified four *C. violaceum* proteins belonging to the OsmC/Ohr family: two paralogues with a high degree of similarity between them, OhrA (CV0209) and OhrB (CV2493) (58% identity), and two hypothetical proteins (CV2756 and CV1324).

**Figure 1:**
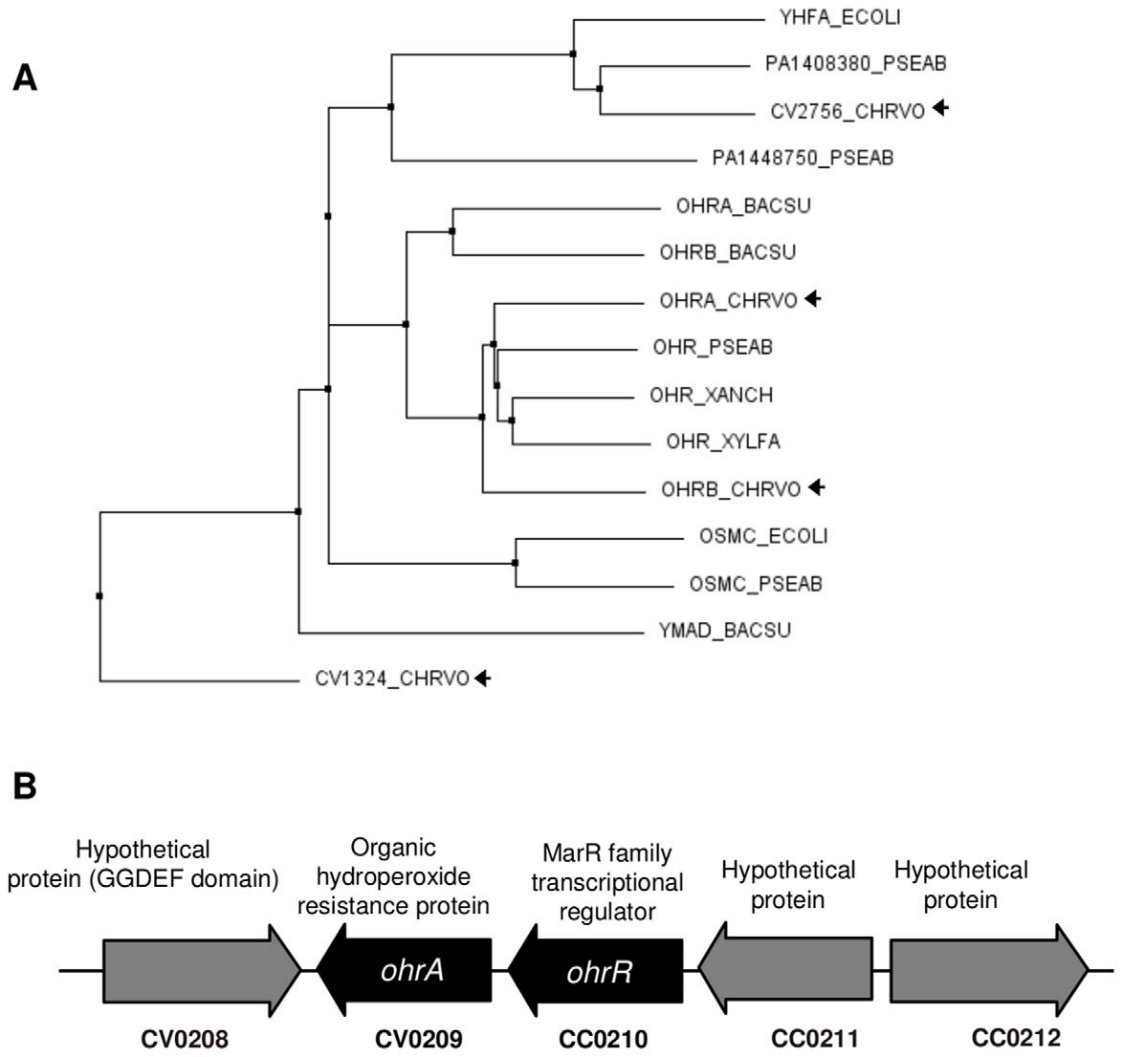
*Chromobacterium violaceum* possesses four proteins belonging to the OsmC/Ohr family. (**A**) Phylogenetic tree of OsmC/Ohr proteins (PF02566) from six selected bacterial genomes. Protein sequences from *Chromobacterium violaceum* (CHRVO), *Pseudomonas aeruginosa* (PSEAE), *Xylella fastidiosa* (XYLFA), *Xanthomonas campestris* pv. *phaseoli* (XANCH), *Bacillus subtilis* (BACSU) and *Escherichia coli* (ECOLI) were aligned using the CLUSTAL W 2.1 program. The tree was obtained by using the neighbor-joining method and Blosum62 from the CLUSTAL W alignment. (**B**) The schematic depicts the chromosomal region of *C. violaceum* around the *ohrA* and *ohrR* genes.

To clarify the relationship among these *C. violaceum* proteins and with other OsmC/Ohr proteins, we performed multiple sequence alignments using the ClustalW algorithm. All aligned proteins contain the two conserved active site cysteines typical of OsmC/Ohr proteins [22]. The phylogenetic tree built from this alignment (Fig. 1A) presents three main branches that fit with the previously described subfamilies, Ohr, OsmC and YhfA [42]. Although a phylogenetic investigation of the Ohr/OsmC proteins was beyond the scope of this work, we could detect significant variation in the number of genes for these proteins per genome and a wide disparity in their distribution after analyzing only six bacterial genomes. Thus, in *C. violaceum*, there are two Ohr proteins (OhrA and OhrB), none in the OsmC branch, one that fits within the YhfA subfamily (CV2756) and one displaying a high divergence in relation to the three subfamilies (CV1324) (Fig. 1A). In several bacteria, *ohr* and *ohrR* are adjacent genes, suggesting that they operate in the same pathway. In the *C. violaceum* genome, next to CV0209 there is a gene encoding for a MarR family regulator (CV0210). Therefore, based on sequence similarity and genome colocalization, it is probable that the adjacent genes CV0209 and CV0210 encode for the OhrA and OhrR proteins, respectively (Fig. 1B).

### OhrA is required for resistance to and the degradation of organic hydroperoxides

**Figure 2:**
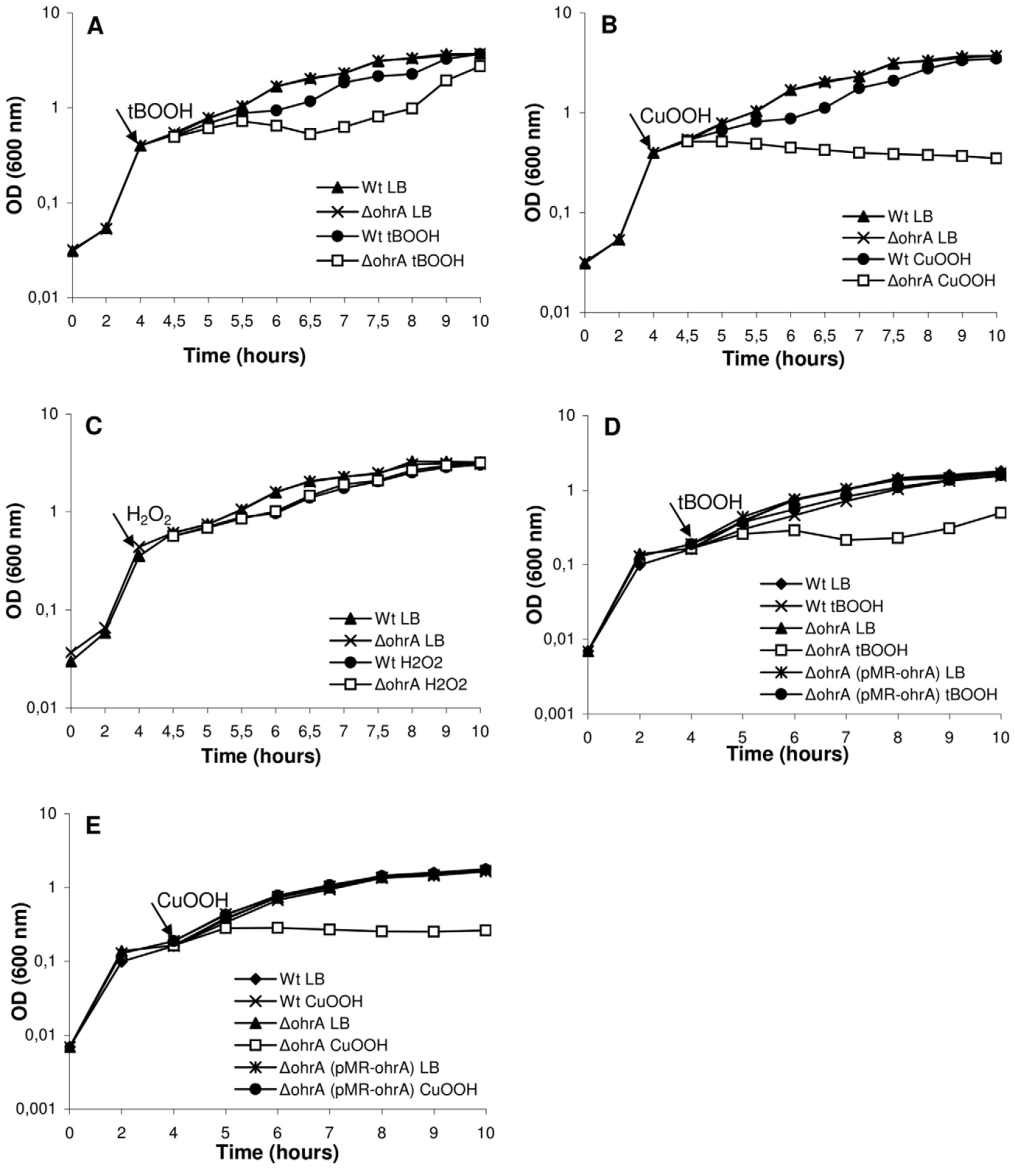
Effect of peroxides on the growth of the wild type and Δ*ohrA* mutant strains. Cultures grown in LB medium for 4 hours (to an OD at 600 nm of 0.4) were divided and either untreated or treated (indicated by arrow) with 140 µM tBOOH (**A**), 60 µM CuOOH (**B**) or 75 µM H_2_O_2_ (**C**). For complementation assays, the indicated strains, harboring the pMR20 vector, were grown in LB medium plus tetracycline for 4 hours, and the cultures were either untreated or treated with 120 µM tBOOH (**D**) or 40 µM CuOOH (**E**). The figure shows a representative experiment of two independent repetitions.

To determine the role of OhrA and OhrR in oxidative stress defense, mutant strains with deletions for the *ohrA* and *ohrR* genes were obtained by allelic exchange, and their growth after oxidant exposure was compared to that of the wild-type strain, ATCC 12472. The *C. violaceum ohrA* deletion mutant was hypersensitive to the organic hydroperoxides *tert*-butyl (tBOOH) and cumene (CuOOH) but not to hydrogen peroxide (Fig. 2A, 2B and 2C). Furthermore, these phenotypes were complemented after providing the *ohrA* gene in *trans* in the plasmid pMR20 (Fig. 2D and 2E). In contrast, the *ohrR* mutant was not sensitive to any of these treatments (data not shown).

**Figure 3:**
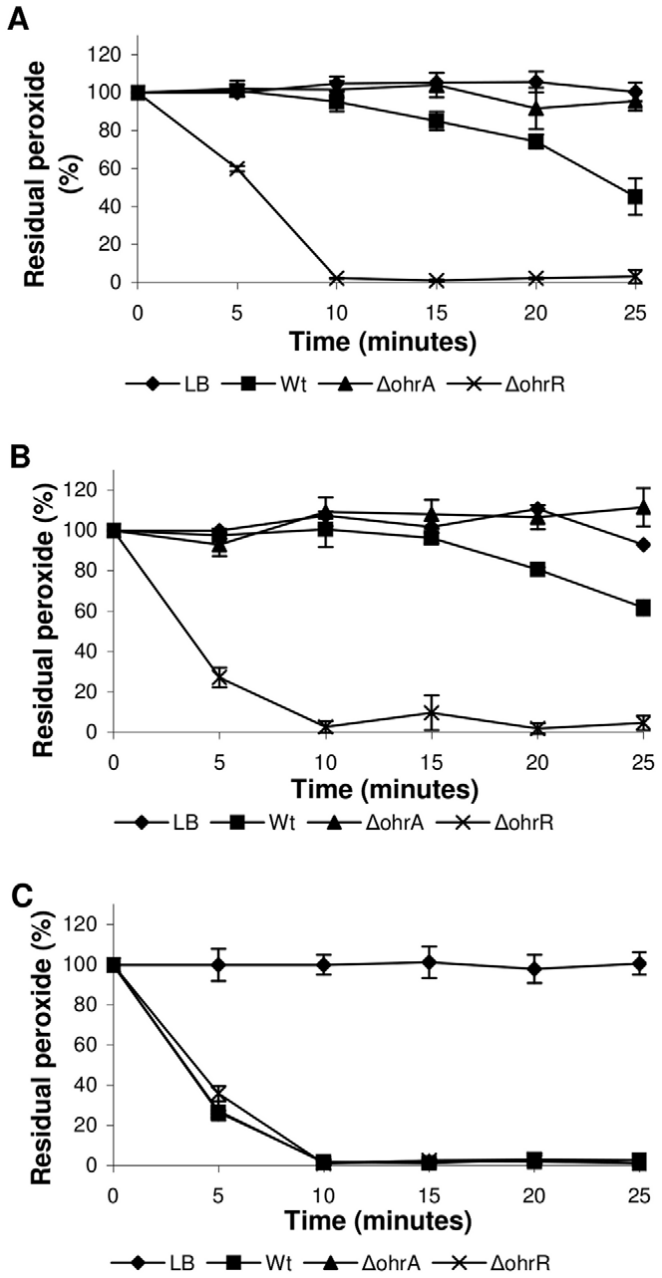
Degradation of peroxides by the *C. violaceum* ATCC 12472 and Δ*ohrA* and Δ*ohrR* mutant strains. Cultures grown in LB medium to an OD at 600 nm of 0.7 were divided and either treated with 300 µM tBOOH (**A**), 200 µM CuOOH (**B**) or 500 µM H_2_O_2_ (**C**). The level of residual peroxides remaining in the culture medium was determined at 5-min intervals using a xylenol orange assay.

To further characterize the relevance of OhrA and OhrR in the response of *C. violaceum* to organic hydroperoxide stress *in vivo*, the peroxide concentration remaining in the culture medium, corresponding to the wild type and mutant strains, was evaluated (Fig. 3). The *ohrA* mutant displayed a markedly decreased capacity to decompose organic hydroperoxides (tBOOH and CuOOH) given that after 25 min the levels of these oxidants remained unaffected, which was similar to the culture not inoculated with bacteria. At the same time, the remaining amount of tBOOH and CuOOH was 50% for the wild type strain. The *ohrR* mutant, in turn, was a more efficient consumer of tBOOH and CuOOH, requiring only 10 min to degrade all the added hydroperoxides (Fig. 3A and 3B). These profiles of organic hydroperoxide consumption suggest that OhrR acts as a negative regulator of *ohrA*. Thus, the *ohrR* mutant cells are probably devoid of repressive regulatory mechanisms, presenting high levels of OhrA. In contrast, both mutant strains degraded H_2_O_2_ similar to the wild type strain, indicating, as expected, that OhrA and OhrR do not participate in the response of *C. violaceum* to this oxidant (Fig. 3C). Taken together, these physiological and biochemical data indicated that OhrA/OhrR is the main pathway for *C. violaceum* to specifically degrade organic hydroperoxides. In contrast to the specificity of OhrA, it has been shown that the pigment violacein produced by *C. violaceum* has a broad antioxidant activity *in vitro* [43].

### Regulation of the *ohrA* and *ohrR* genes in response to stresses

**Figure 4:**
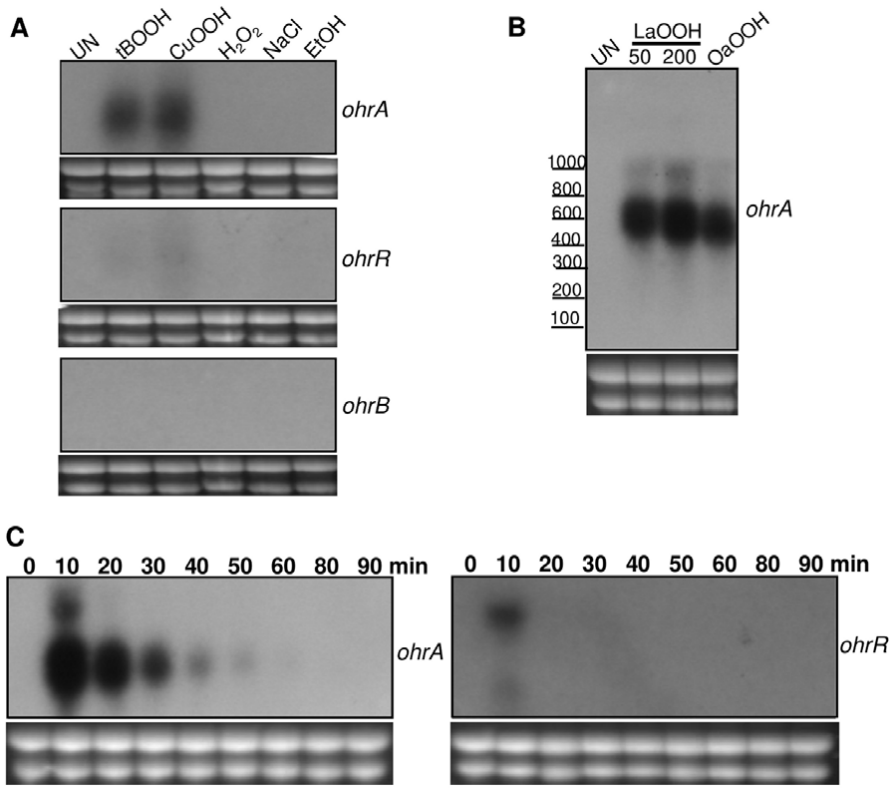
Northern blot analysis of the *C. violaceum ohr* genes. (**A**) Total RNA was extracted from exponential-phase cultures of wild type ATCC 12472 strain under uninduced conditions (UN) and after exposure to 200 µM tBOOH, 200 µM CuOOH, 200 µM H_2_O_2_, 3% NaCl and 4% ethanol (EtOH) for 15 min. RNA was separated on 1.5% agarose gel, transferred to a nylon membrane and hybridized with radiolabeled probes specific to *ohrA*, *ohrR* or *ohrB*. (**B**) *ohrA* induction by lipid hydroperoxides. Wild type cells were exposed to 50 µM and 200 µM linoleic acid hydroperoxide (LaOOH) or to 50 µM oleic acid hydroperoxide (OaOOH) for 15 min prior to Northern blot. (**C**) Time course of *ohrA* and *ohrR* induction. RNA samples from wild type cells untreated (0) or treated with 100 µM tBOOH for the indicated times (0 to 90 min) were hybridized with probes specific to *ohrA* or *ohrR*. Ethidium bromide staining of rRNA was used as a loading control and is shown below each Northern blot experiment. The sizes of the bands were estimated by ethidium bromide staining of the molecular weight marker Low Range RNA Ladder Riboruler (Fermentas).

To further evaluate the physiological roles of OhrA/OhrR in the response of *C. violaceum* to organic hydroperoxide insults, the expression profiles of the *ohrA*, *ohrB* and *ohrR* genes were investigated by Northern blot using exponentially growing cells treated with several stressful conditions (Fig. 4). We found that *ohrA* was markedly induced by the synthetic organic hydroperoxides CuOOH and tBOOH but was not affected by H_2_O_2_, ethanol or NaCl treatments (Fig. 4A). Similarly, the *ohrR* gene displayed organic hydroperoxide-specific expression (tBOOH and CuOOH), but the induction ratios were lower than those observed for the *ohrA* gene. When the same RNA preparations were probed with an *ohrB*-specific probe, no transcript was detected in any of the examined conditions (Fig. 4A). The induction of *ohrA* and *ohrR* expression by organic hydroperoxides has been described before [14]–[16], whereas *ohrB* induction by ethanol and NaCl, observed in other bacteria [13], [15], was not verified here in *C. violaceum*.

To further examine the effect of more complex organic hydroperoxides in *ohrA* expression, Northern blot analyses were performed using RNA samples from wild type cells exposed to linoleic acid hydroperoxide and oleic acid hydroperoxide (Fig. 4B). Even when used at lower concentrations (50 µM), these lipid hydroperoxides were more potent inducers of *ohrA* expression than tBOOH and CuOOH (Fig. 4A and 4B). Under these conditions, a longer transcript of approximately 1.1 kb in length, which is equivalent to the expected size for bicistronic *ohrR*-*ohrA* mRNA, was also detected in addition to the 0.5 kb monocistronic *ohrA* transcript. The longer bicistronic *ohrR-ohrA* transcripts were less abundant than the shorter monocistronic transcripts of *ohrA* (Fig. 4B). In *X. campestris* and *P. aeruginosa*, where the *ohrR* and *ohrA* genes are also oriented in a head-to-tail manner, as observed in *C. violaceum* (Fig. 1B), similar transcriptional organizations were also observed [16], [44]. Then, we examined the time course induction of *ohrA* and *ohrR*. Both genes displayed transient induction because their mRNA levels were elevated 10 min after cell treatment with 100 µM tBOOH, but these levels decreased, reaching the prestimulus condition in a few minutes (for *ohrR*) and in about one hour (for *ohrA*) after the treatment (Fig. 4C).

**Figure 5:**
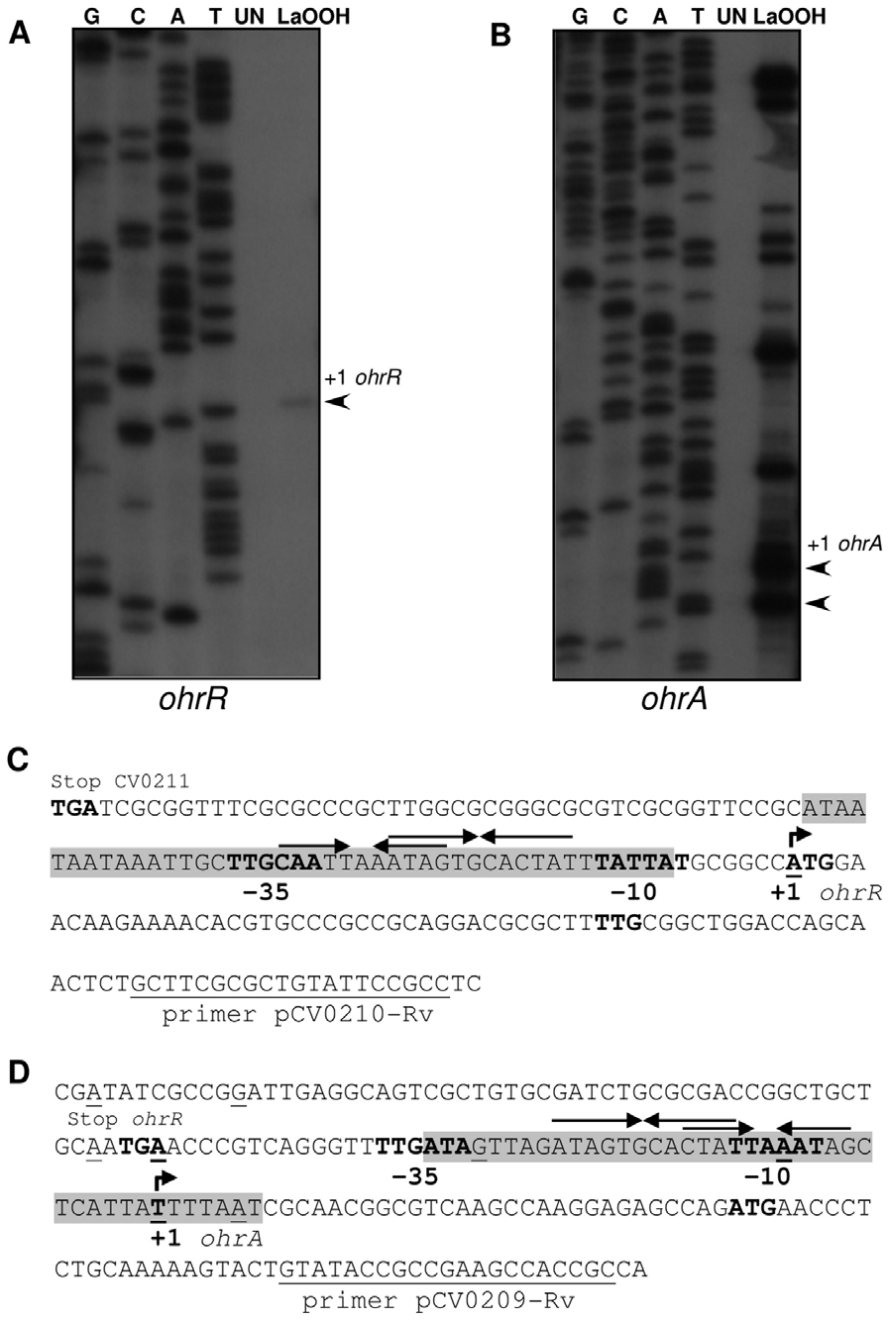
Mapping transcription start sites for *orhR* and *ohrA*. (**A and B**) Primer extension analysis of *ohrR* (**A**) and *ohrA* (**B**) using RNA extracted from *C. violaceum* cultures either untreated (UN) or treated with 200 µM linoleic acid hydroperoxide (LaOOH) for 15 min. The Sanger sequencing ladders (G, C, A, T) were generated using the same primers as those used for the primer extension. (**C and D**) Sequences of the *ohrR* (**C**) and *ohrA* (**D**) promoters. The putative −10 and −35 promoter elements, the transcription start sites (+1, bent arrows), the stop codons, and the putative *ohrR* and *ohrA* translation initiation codons (ATG) are shown in bold. The locations of the multiple primer extension products observed for *ohrA* are indicated in the *ohrA* promoter sequence (underlined nucleotides). The DNase I-protected regions for OhrR binding are shaded, and the inverted repeat motifs are indicated by arrows. The primers used in primer extension assays are indicated.

Next, primer extension experiments were performed on RNA samples from uninduced (UN) and linoleic acid hydroperoxide (LaOOH)-induced samples to locate the *ohrR* and *ohrA* transcription start sites (Fig. 5). When using a specific primer to *ohrR*, the results showed that the single primer extension product detected (Fig. 5A) corresponded to the *ohrR* transcription start site (+1), located at the A residue coinciding with the translation initiation codon (ATG) of *ohrR*. The putative *ohrR* promoter elements were predicted (TTGCAA- 17 nucleotides -TATTAT) and match quite well with the consensus sequence recognized by the primary *E. coli* σ^70^ factor (Fig. 5C). Based on a multiple sequence alignment, we reannotated the OhrR translation initiation codon (an ATG codon instead of the previously annotated TTG codon, Fig. 5C). Although, as a consequence, an *ohrR* leaderless mRNA is now predicted, several lines of evidence support this assumption: (i) The amino terminal region of the deduced OhrR protein matches with that of other OhrR proteins, (ii) the position of the conserved residues Cys21 and Cys126 maintain a good agreement with the 2-Cys OhrR subfamily and (iii) in other bacteria, such as *X. campestris* and *S. coelicolor*, *ohrR* transcription also initiates at the A residue of the ATG translation initiation codon [15], [45].

When a primer extension analysis was performed with the *ohrA* gene, several primer extension products, strongly induced by LaOOH, were detected (Fig. 5B). These products appear to be specific to *ohrA* because they were detected under diverse experimental conditions, changing annealing temperatures and RNA primer ratios (data not shown). The primer extension products mapped to the intergenic region between *ohrR* and *ohrA* and also within the *ohrR* gene (underlined nucleotides, Fig. 5D). The *ohrA* transcription start site (+1) is probably the T residue located 36 nucleotides upstream from the translation initiation codon because its position is consistent with transcription being driven from the σ^70^ type promoter (TTGATA- 16 nucleotides -TTAAAT) identified in the sequence. The other products could be alternative transcription start sites or, more probably, correspond to several 5′ ends of the *ohrA* mRNA, processed from the bicistronic *ohrR-ohrA* mRNA detected by Northern blot (Fig. 4B and 4C), similar to what has been observed with *X. campestris* [44].

### OhrR binds to *ohrA* and *ohrR* promoters and its binding is inhibited by peroxides

**Figure 6:**
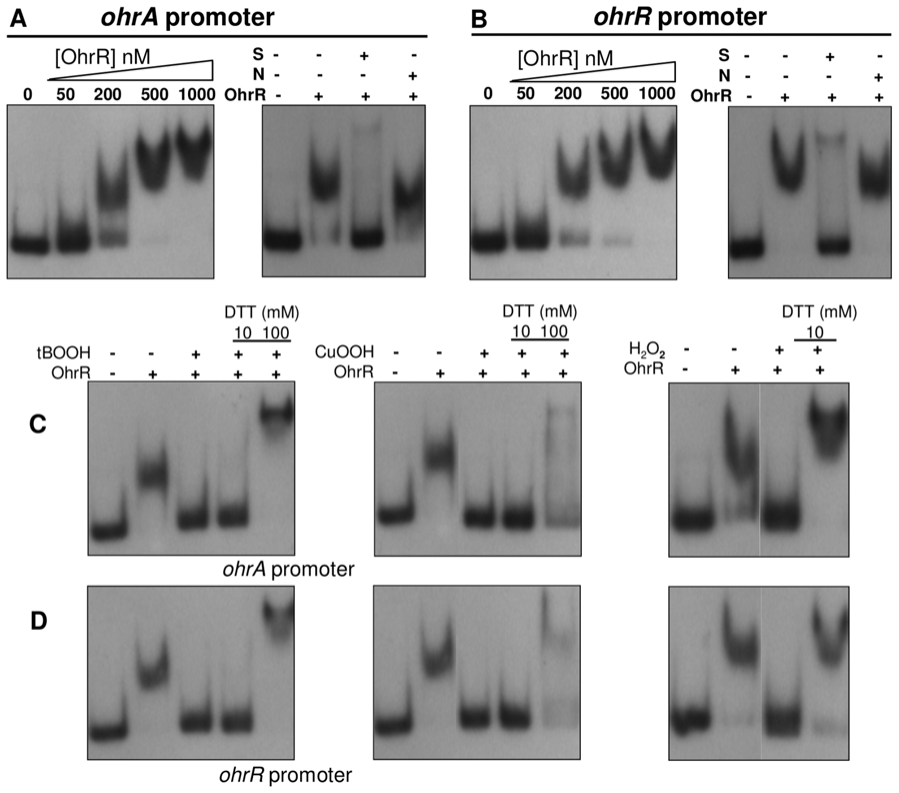
Interaction of OhrR with the promoter of *ohrA* and *ohrR*, as determined by EMSA. Labeled probes containing the promoter region of *ohrA* (**A**) and *ohrR* (**B**) were incubated with increasing concentrations of purified OhrR protein, as indicated, and the mixture was separated in a polyacrylamide non-denaturing gel. Competition assays **(right panels)** using 250 nM OhrR were performed in the presence of 30-fold excess of unlabeled fragments of the same region (S) or the *ohrR* coding region (N) as competitors. (**C and D**) Effects of oxidants and DTT on OhrR binding. EMSA assays using OhrR with the *ohrA* promoter (**C**) or *ohrR* promoter (**D**). ^32^P-Labeled probes were incubated with 250 nM purified OhrR. Either 0.3 mM *tert*-butyl hydroperoxide (tBOOH), 0.3 mM cumene hydroperoxide (CuOOH) or 1 mM H_2_O_2_ was added to the binding reaction and incubated for 10 min at 25°C. When indicated, DTT was then added into the reactions, and incubation continued at 25°C for 15 min.

The direct interaction of OhrR with the *ohrA* and *ohrR* promoters was tested by electrophoretic mobility shift assays (EMSA) using recombinant *C. violaceum* OhrR and labeled DNA fragments. The addition of various concentrations of purified OhrR resulted in a slower migration of the probes in a dose-dependent manner, indicating that OhrR directly binds to the *ohrA* and *ohrR* promoters *in vitro* (Fig. 6A and 6B). Competition assays using specific and non-specific unlabeled DNA confirmed the specificity of OhrR binding (Fig. 6A and 6B). In contrast, the OhrR protein did not bind to the promoter region of *ohrB* as well as to an unspecific probe even at a 1000 nM concentration. Nonspecific complexes between OhrR and DNA began to form at concentrations above 2500 nM (Fig. S1).

The EMSA assay was also used to test the *in vitro* effects of oxidants on the ability of OhrR to bind to the *ohrA* and *ohrR* promoters. The addition of tBOOH, CuOOH and H_2_O_2_ to the binding reaction lead to the loss of OhrR binding to its target sites, although a higher concentration of H_2_O_2_ was required for OhrR inactivation. Binding activity was recovered upon the addition of the reducing agent dithiothreitol (DTT) (Fig. 6C and 6D). These results indicate that OhrR oxidation renders it unable to bind to the *ohrA* and *ohrR* promoters *in vitro*, but the oxidized protein could be re-reduced again by DTT (100 mM), recovering its DNA-binding activity. Similarly, it has been reported in *B. subtilis* and *P. aeruginosa* that OhrR activity was abolished after exposure to several oxidants *in vitro* [26], [46].

### Mapping of OhrR-binding sites within the *ohrA* and *ohrR* promoters

**Figure 7:**
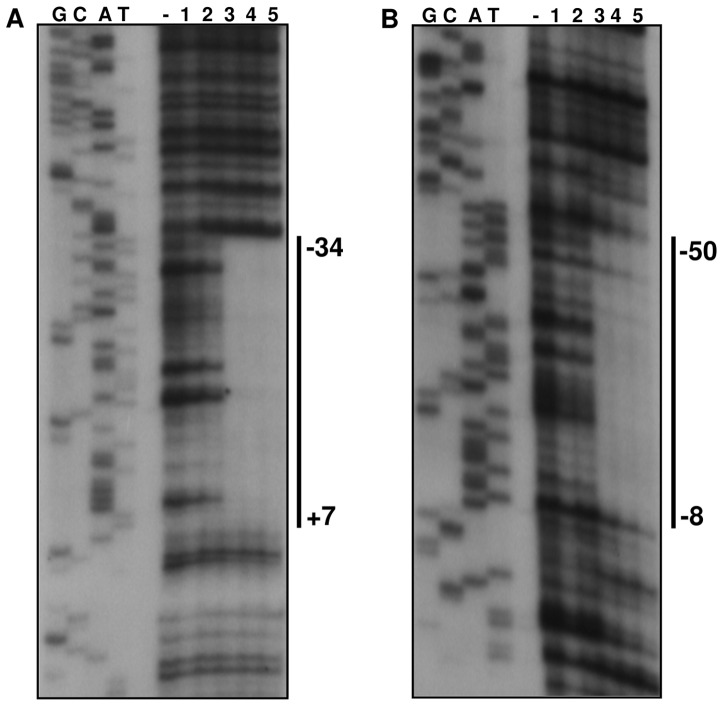
Mapping of OhrR-binding sites on the *ohrA* and *ohrR* promoters. DNase I footprinting analyses were performed using probes covering the *ohrA* (**A**) and *ohrR* (**B**) promoter regions and increasing concentrations of purified OhrR protein (10, 50, 250, 500 and 1000 nM, lanes 1 to 5). The minus sign (−) represents the probe fragments treated with DNase I in the absence of OhrR. The regions protected by OhrR are indicated by a solid line and numbered according to transcription start sites (+1). The samples were run in parallel with the sequencing ladders (G, C, A, T), which were generated using the promoter fragments themselves as a template.

To determine the precise localization of the OhrR-binding sites within the *ohrA* and *ohrR* promoter regions, we performed DNase I footprinting experiments. The region protected from DNase I digestion on the *ohrA* promoter fragment spans from nucleotides +7 to −34, with respect to the *ohrA* transcriptional start site (Fig. 7A). On the *ohrR* promoter fragment, OhrR binding resulted in an area of protection extending from nucleotides −8 to −50, relative to the *ohrR* transcription start site (Fig. 7B). As shown in Fig. 5C and 5D, the OhrR-binding sites overlap to the −10 and −35 elements of the *ohrR* and *ohrA* promoters, an arrangement consistent with the repressor function of OhrR. A comparison of the two protected regions allowed for the identification of an identical 12-nucloetide sequence (ATAGTGCACTAT), which is a perfect inverted repeat (Fig. 5C and 5D). Although this sequence could be the operator site of OhrR, other imperfect inverted repeat sequences were also detected (CTATT-A-AATAG for OhrA and CAATT-A-AATAG for OhrR) that share more similarity with the putative OhrR binding sequences of other bacteria, including the core sequence AATT-N-AATT [14]–[16], [26], [45]. Taken together, the gel shift and DNase I footprinting results demonstrate that reduced OhrR binds with a similar affinity to specific operator sites on the *ohrA* and *ohrR* promoters and also reinforces the finding that *ohrA* and *ohrR* have distinct transcription start sites (Fig. 5).

### Sensing of organic hydroperoxides by OhrR requires the conserved Cys21 residue

**Figure 8:**
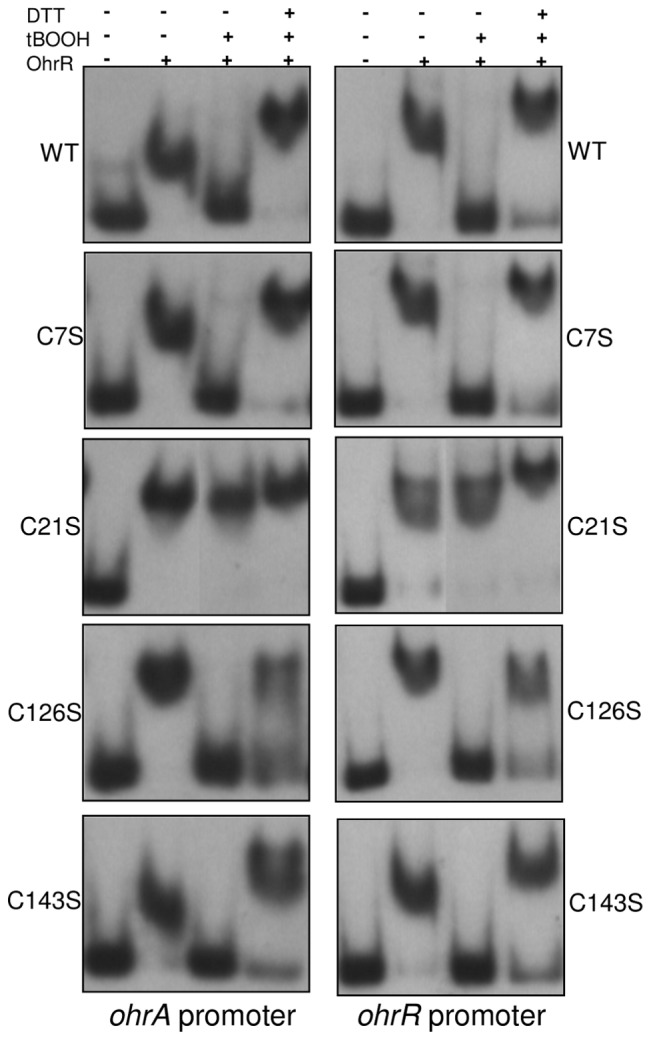
Effect of OhrR cysteine mutations on peroxide sensing *in vitro*. EMSA assays were performed using the labeled probes (*ohrA* or *ohrR* promoters) incubated with 250 nM OhrR WT, OhrR C7S, OhrR C21S, OhrR C126S and OhrR C143S purified proteins. When indicated, 0.5 mM *tert*-butyl hydroperoxide (tBOOH) or 0.5 mM tBOOH plus 100 mM DTT were added in the binding reactions.

When considering our reannotation, the OhrR from *C. violaceum* contains four cysteines, including the fully conserved residue at position 21 (Cys21) and the conserved residue at position 126 (Cys126), which are typical of OhrR proteins belonging to the 2-Cys subfamily [11]. To determine whether these cysteine residues play a role in redox sensing *in vitro*, we generated OhrR C7S, C21S, C126S and C143S site-directed mutant proteins. All recombinant proteins were expressed, purified and investigated through EMSA assays with labeled probes corresponding to the *ohrA* and *ohrR* promoters (Fig. 8). Under reducing conditions, the wild type and four OhrR mutant proteins were able to bind to both DNA probes. The addition of the oxidant tBOOH abolished the binding activity of the OhrR wild type, C7S, C126S and C143S proteins. The capacity of these four proteins to bind DNA was recovered upon the addition of DTT, although for the C126S protein this recovery was only partially achieved. However, the DNA-binding activity of the C21S mutant protein was not affected by the tBOOH and DTT treatments (Fig. 8). These data indicate that the conserved Cys21 residue is required for OhrR to sense organic hydroperoxide *in vitro*.

**Figure 9:**
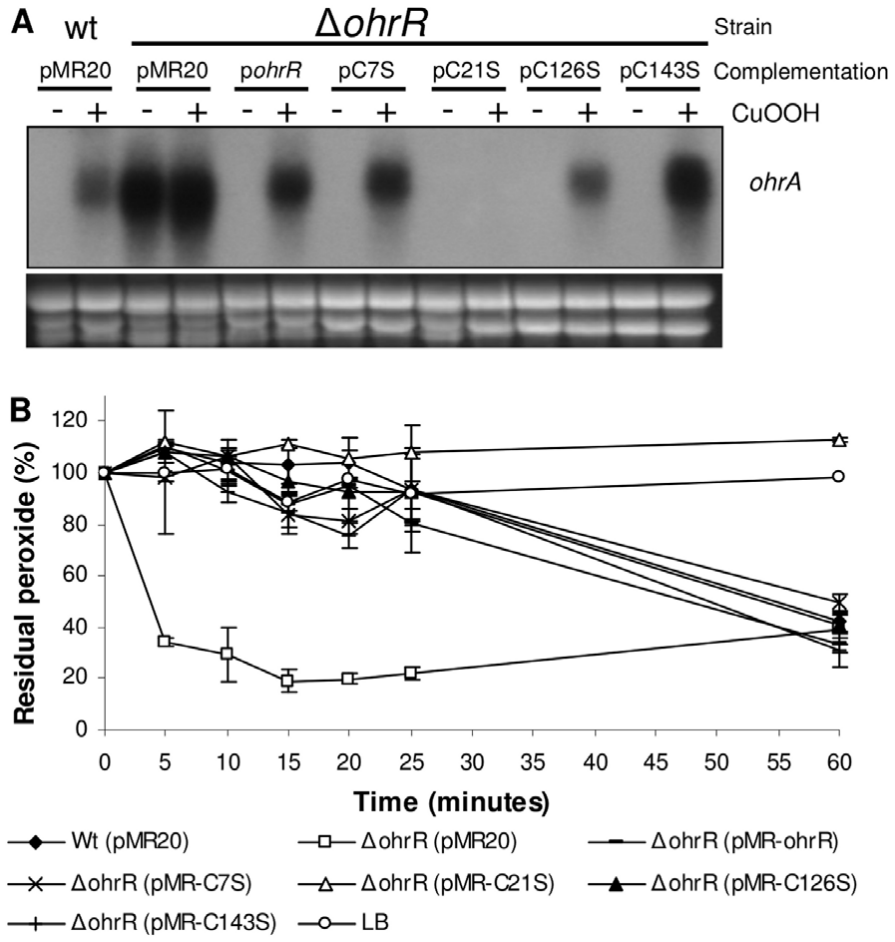
The essential role of OhrR Cys21 in cumene hydroperoxide sensing *in vivo*. (**A**) Cultures of wild type (ATCC 12472) and Δ*ohrR* mutant strains containing empty vector (pMR20) or the Δ*ohrR* mutant strain complemented with OhrR, OhrR C7S, OhrR C21S, OhrR C126S and OhrR C143S were either untreated or treated with 200 µM cumene hydroperoxide (CuOOH). The expression of *ohrA* was monitored by Northern blot. Ethidium bromide staining of rRNA was used as a loading control. (**B**) Effect of OhrR cysteine mutations on the degradation of cumene hydroperoxide. Cultures of the indicated strains were growth in LB medium with 10 µg/ml tetracycline. CuOOH (200 µM) was then added, and the level of residual peroxide was determined using the xylenol orange assay.

To evaluate the relevance of the four OhrR cysteine residues *in vivo*, after site-directed mutagenesis, the mutated OhrR genes were cloned into the expression vector pMR20. Then, the empty vector and the constructs pMR-*ohr*, pMR-C7S, pMR-C21S, pMR-C126S and pMR-C143S were introduced into the *ohrR* mutant strain by conjugation. The wild type strain, ATCC 12472, and the *ohrR* mutant complemented with these different OhrR variants were grown under uninduced and CuOOH-induced conditions, and their levels of *ohrA* mRNA were measured by Northern blot (Fig. 9A).

As expected, *ohrA* expression was induced by CuOOH in the wild type strain. High levels of the *ohrA* mRNA were detected in the *ohrR* mutant regardless of whether or not CuOOH was present, indicating that OhrR acts as a transcriptional repressor of *ohrA* expression (Fig. 9A). The capacity of the diverse OhrR variants to function as repressors or sensors in response to organic peroxide was evaluated in the *ohrR* mutant background. In the uninduced condition, the proteins C7S, C21S, C126S and C143S allowed for the complementation of the *ohrR* mutant strain because they were able to repress *ohrA* expression similar to the wild type OhrR, indicating that the cysteines have no role in the binding of OhrR to the *ohrA* promoter (Fig. 9A). Otherwise, upon exposure to CuOOH, only the OhrR wild type, C7S, C143S and C126S (in this case only partially) allowed for *ohrA* derepression, whereas cells expressing the C21S protein were insensitive to CuOOH treatment. These results indicate that Cys21 is also critical for organic hydroperoxide sensing by OhrR *in vivo*.

Extending this rationale to enzymatic activity, we performed peroxide consumption assays using the same bacterial strains to test the ability of the OhrR mutant proteins to complement the *ohrR* mutant strain (Fig. 9B). As expected, the *ohrR* mutant degraded CuOOH more efficiently than the wild type strain, as previously verified (Fig. 3 versus Fig. 9B). The *ohrR* mutant strains complemented with the C7S, C126S, C143S and wild type OhrR degraded CuOOH similarly to wild type strain (Fig. 9B). However, when the complementation was performed using the C21S protein, the resultant strain displayed a hydroperoxide degradation profile comparable to that verified for the *ohrA* mutant strain (Fig. 3 versus Fig. 9B). This result supports the notion that the C21S protein no longer responds to oxidants and persists bound to the *ohrA* promoter even in the presence of the organic hydroperoxide.

### OhrR forms intermolecular disulfide bonds upon oxidation

**Figure 10:**
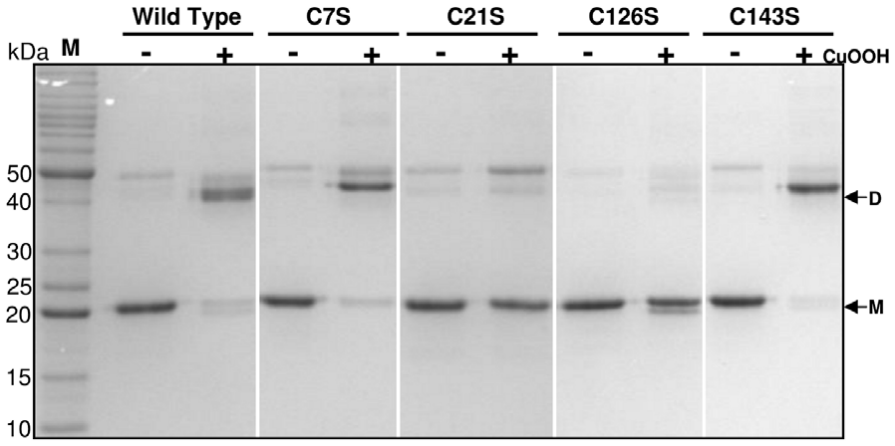
Investigation of intermolecular disulfide bond formation on OhrR by nonreducing SDS-PAGE. Purified OhrR variants were reduced by DTT treatment. Samples of reduced proteins were either untreated (−) or treated (+) with 100 µM cumene hydroperoxide (CuOOH) for 30 min before alkylation. The NEM-alkylated proteins were separated by nonreducing SDS-PAGE, stained with Coomassie Brilliant Blue and subsequently destained. The monomeric (M) and dimeric (D) forms of OhrR are indicated by arrows. M represents the protein molecular mass standards.

To further investigate the mechanism by which the *C. violaceum* OhrR protein is oxidized, we performed a nonreducing SDS-PAGE analysis using purified OhrR variants (Fig. 10). When the wild type OhrR containing all four cysteine residues was reduced by DTT treatment, it migrated as a monomer of approximately 20 kDa (the calculated molecular mass of the His-OhrR is 20.87 kDa). After oxidation by CuOOH, most of the protein was converted to covalent dimers (40 kDa). This dimerization upon cumene oxidation was also observed for the C7S and C143S mutant proteins, suggesting that Cys7 and Cys143 have no role in dimer formation. Conversely, the C21S and C126S proteins remained predominantly as monomers even under CuOOH oxidation (Fig. 10). These results indicate that Cys21 and Cys126 form an intermolecular disulfide bond when OhrR is oxidized by cumene, similar to what has been observed with *X. campestris* [29].

### OhrR is specifically reduced by the thioredoxin system in vitro

**Figure 11:**
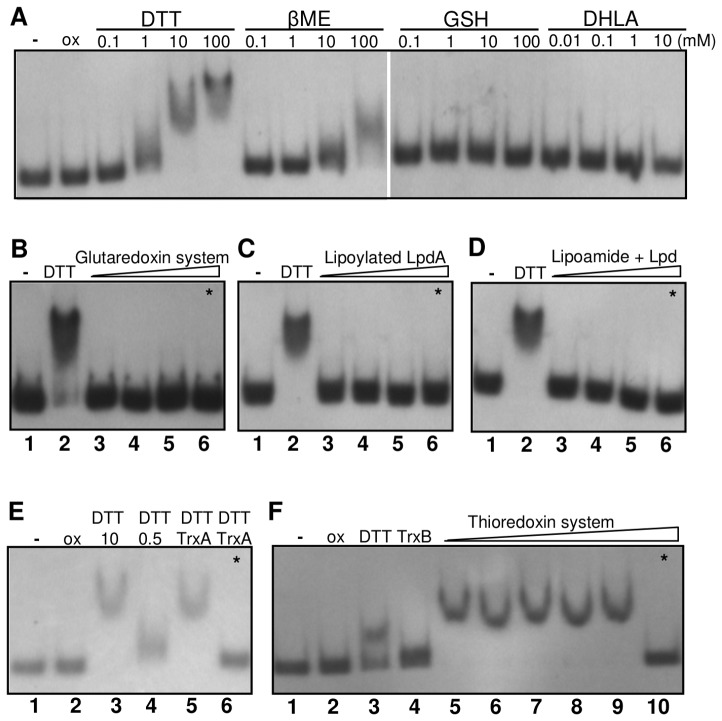
Effect of thiol systems on OhrR reduction *in vitro*, as monitored by EMSA. Samples of purified *C. violaceum* OhrR (5 µM air-oxidized protein) were treated with the indicated reductants for 30 min at 30°C. All lanes contain the *ohrA* promoter region as a probe and 250 nM OhrR except lanes 1 (−), which contain the DNA probe only, or lanes 6 and 10 (*), where OhrR was omitted in the reaction mixtures. (**A**) Oxidized OhrR (ox) treated with increasing concentrations of the low molecular weight thiols dithiothreitol (DTT), β-mercaptoethanol (βME), reduced glutathione (GSH) and dyhydrolipoic acid (DHLA). (**B**) Oxidized OhrR incubated with 0.5 mM NADPH, 10 µg/ml glutathione reductase (GR), 10 mM glutathione (GSH) plus 1, 10 and 20 µM glutaredoxin 3 (GrxC) (lanes 3–5). (**C**) Oxidized OhrR incubated with 0.5 mM NADH plus 1, 10 and 50 µM LpdA (lanes 3–5). (**D**) Oxidized OhrR incubated with 0.5 mM NADH, 10 µM lipoamide plus 1, 5 and 10 µM dihydrolipoamide dehydrogenase (Lpd) (lanes 3–5). Lanes 2 in panels B, C and D contain OhrR treated with 10 mM DTT (positive controls). (**E**) Oxidized OhrR (lane 2) was incubated with 10 mM DTT (lane 3), 0.5 mM DTT (lane 4), and 0.5 mM DTT plus 10 µM thioredoxin (TrxA) (lane 5). (**F**) Samples of oxidized OhrR (lane 2) were incubated with 1 mM DTT (lane 3); 0.5 mM NADPH plus 5 µM thioredoxin reductase (TrxB) (lane 4); 0.5 mM NADPH, 1 µM TrxB plus 10, 20 and 40 µM TrxA (lanes 5–7); 0.5 mM NADPH, 5 µM TrxB plus 20 and 40 µM TrxA (lanes 8–9). The control reactions without OhrR to confirm the absence of unspecific DNA binding by the enzymes (lanes 6 and 10, indicated by an asterisk) contain the same mixtures of the samples in lanes 5 and 9 of each respective panel.

Taking into account that only the reduced form of OhrR binds DNA and that the oxidized protein is re-reduced by DTT treatment (Fig. 6), we monitored the redox dependent ability of OhrR to bind DNA by EMSA assays, replacing DTT with several thiol reductants in the binding buffer (Fig. 11). When oxidized OhrR (ox) was treated with increasing concentrations of several low molecular weight (LMW) thiols, besides DTT (1–10 mM), only β-mercaptoethanol (βME) was effectively able to reduce OhrR, but at higher concentrations (100 mM). Both DTT and βME are synthetic thiols. The biological low molecular weight thiols glutathione (GSH) and dyhydrolipoic acid (DHLA) failed to reduce OhrR because no OhrR binding to the *ohrA* promoter probe was observed (Fig. 11A).

Thus, to investigate whether OhrR could be regenerated by enzymatic systems, we reconstituted the components of three important biological reducing pathways (the glutaredoxin, thioredoxin and lipoamide systems) *in vitro* using purified heterologous recombinant proteins (Fig. 11B–F). All three reducing systems presented disulfide reductase activity, as confirmed by DTNB assays (Fig. S2). However, when oxidized OhrR was incubated with glutaredoxin 3 from *Xylella fastidiosa* in the presence of NADPH, glutathione reductase and glutathione, no OhrR binding was observed (Fig. 11B). Similarly, neither the lipoylated enzyme LpdA from *X. fastidiosa* (Fig. 11C) nor lipoamide reduced by dihydrolipoamide dehydrogenase (Lpd) (Fig. 11D) provoked recovery of the OhrR-binding activity. On the other hand, OhrR reduction by DTT (0.5 mM) was stimulated by thioredoxin (TrxA) from *E. coli* (Fig. 11E, lane 5), suggesting that the thioredoxin system could be an enzymatic reductant for OhrR. In fact, when the complete thioredoxin system (TrxA in the presence of TrxB and NADPH) was used to replace DTT (Fig. 11F), we verified the recovery of the OhrR- binding ability to the *ohrA* promoter (Fig. 11F, lanes 5–9). These biochemical results indicate that the *C. violaceum* OhrR is specifically reduced by thioredoxin, among other biological reducing systems (glutaredoxin and lipoamide).

## Discussion

Reversible thiol oxidation plays a key role in controlling the activity of bacterial regulators that operate as direct thiol-based redox switches [11], [47]. The inactivation pathways of OhrR regulators under organic hydroperoxide stress include both the formation of intermolecular disulfide bonds, as described for the 2-Cys OhrR in *X. campestris* [29] as well as the formation of mixed disulfides with LMW thiols, as reported for the 1-Cys OhrR in *B. subtilis* [27]. As more is learned about OhrR systems, the diversity of their mechanisms is revealed. Indeed, although the majority of OhrR proteins repress their target genes when bound to the cognate DNA, in the case of *S. coelicolor*, the reverse is observed for the corresponding protein bound to *ohrR* promoter [15]. Therefore, our description of the mechanism by which the OhrR/OhrA proteins from *C. violaceum* sense and respond to organic hydroperoxide insults contributes to our understanding of this system as a whole.

Except under harsh oxidative conditions that result in irreversible overoxidation [48], OhrR proteins appear to function as reversible regulators, a required feature for a biological redox switch. Therefore, it is reasonable to propose that bacteria have unidentified pathways for the regeneration of active OhrR by thiol-disulfide exchange reactions. In the present work, we have presented evidence that thioredoxin is a natural reducing system for OhrR in the human opportunistic pathogen *C. violaceum*. Additionally, our phenotypic, genetic and biochemical data indicate that OhrR/OhrA is the main dedicated system for the sensing and detoxification of organic hydroperoxides, at least under the conditions investigated here. These novel findings lead us to propose the following tentative model that combines transcriptional regulation and control of OhrR activity by disulfide bond formation with subsequent reduction via the thioredoxin system.

Under unstressed conditions, reduced OhrR binds to operator sites on *ohrA* and *ohrR* promoters with similar affinities (Figs. 6, 7, 8), repressing the transcription of both genes, probably by blocking the access of RNA polymerase because the OhrR-binding sites overlap the promoter elements (Fig. 5) and no transcription was observed under basal (reducing) conditions (Fig. 4). Exposure to organic hydroperoxide causes de-repression of both genes, but the *ohrA* monocistronic transcripts are much more abundant than the *ohrR* monocistronic transcripts or the bicistronic transcripts (Fig. 4). Based on the primer extension and Northern blot data, we propose that this distinct expression profile is a consequence of the differential strength of the *ohrA* and *ohrR* promoters (with individual transcription start sites) and also probably due to processing of the bicistronic transcripts. Despite minor differences, this hypothesis is in good agreement with the transcriptional regulation of *ohrR-ohr* in *X. campestris* [44] and *P. aeruginosa* [16] and is also consistent with the EMSA (Fig. 6) and DNase I footprinting assays (Fig. 7), which indicated that OhrR can bind to the two promoters. When considering that the *ohrR* transcription start site overlaps with the translation initiation codon (Fig. 5C), we suggest that the *ohrR* leaderless mRNA might be inefficiently translated, which is similar to the situation observed for the *ohrR* in *X. campestris* [45]. Together, these transcriptional and post-transcriptional features would result in a significantly higher production of OhrA compared to that of OhrR.

OhrR inactivation initially proceeds by Cys21 oxidation to a sulfenic acid (Cys-SOH), followed by a condensation reaction with Cys126-SH (Figs. 8 and 10), giving rise to an intermolecular disulfide, as described for the 2-Cys OhrR in *X. campestris* [29]. Although in *X. campestris* both cysteine residues are essential for redox sensing [29], our *in vitro* and *in vivo* analyses using OhrR proteins mutated at each cysteine residue indicated that Cys21 is critical for OhrR sensing (Figs. 8 and 9), while Cys126 appears to have a role in preventing overoxidation (Fig. 10), which is similar to the *P. aeruginosa* OhrR [16]. This is because in non-reducing SDS-PAGE, OhrR intermolecular disulfide is expected to migrate as a dimer; whereas reduced (-SH) and overoxidized forms (-SO_2_H^−^;-SO_3_H^−^) are expected to migrate as monomers. Finally, we determined that the oxidized OhrR is specifically reduced *in vitro* by thioredoxin (Fig. 11), suggesting that this reducing system could contribute to the regeneration of the reduced state of the OhrR, thereby shutting off the response. Consistent with this interpretation, the expression of *ohrA* and *ohrR* in *C. violaceum* (Fig. 4C) and in *S. coelicolor* [15] displayed rapid induction kinetics after *tert*-butyl exposure and returned to basal levels a few min later. Moreover, it has been described in *Agrobacterium tumefaciens* that the expression of *trxA*, a gene encoding for thioredoxin, is induced by organic hydroperoxide [49].

Although we can not exclude the existence of other reducing pathways for OhrR, the thioredoxin system appears to be quite specific because neither the glutathione/glutaredoxin system nor lipoylated enzymes used here were able to reduce OhrR *in vitro* (Fig. 11). Inspection of the *C. violaceum* genome sequence allowed for the detection of genes encoding for the proteins for all three reducing systems. In particular, with respect to the thioredoxin system, we found three probable thioredoxins (CV1584, CV1106 and CV1895) and one thioredoxin reductase (CV2813). The heterologous *E. coli* thioredoxin system that we used here for OhrR reduction *in vitro* appears to be appropriate when considering that the *C. violaceum* TxrA (CV1584) and TrxB (CV2813) proteins share 74% and 68% amino acid identities with their corresponding proteins in *E. coli*. Whether the thioredoxin system is able to reduce OhrR *in vivo* remains an interesting open question. Curiously, it has recently been described that enzymes of the Ohr/OsmC family, such as the Ohr of *X. fastidiosa*, OsmC of *E. coli* [23] and Ohr of *M. smegmatis* [50], are specifically reduced by lipoylated proteins, suggesting that although belonging to the same route of organic hydroperoxide detoxification, OhrR and Ohr employ different cellular reducing pathways. Interestingly, OxyR (a well-established hydrogen peroxide sensor) is specifically reduced by glutaredoxin, another thiol-reducing enzyme [30], which probably reflects distinct response pathways for hydrogen and organic hydroperoxides. The specificity of redox signaling pathways is a theme that is receiving growing attention in both bacteria and eukaryotes [51]–[53].

In contrast with the well-established role of Ohr proteins in organic hydroperoxide defense under *in vitro* conditions, little is known about the contribution of this system in bacterial pathogenicity. Although previous work has determined that the *ohr* gene is induced *in vivo* during the *Actinobacillus pleuropneumoniae* infection of pigs [54], it was only very recently that Ohr was implicated as a critical virulence determinant required for the intracellular pathogen *Francisella tularensis* to infect macrophages and mice [55]. In *P. aeruginosa*, OhrR, but not Ohr, contributes to virulence in a *Caenorhabditis elegans* pathogenicity model [16]. The fact that *C. violaceum* is an opportunistic pathogen combined with the recent establishment of pathogenicity models in *C. elegans* [37], mouse and several mammalian cell lines [38] raises the perspective for future investigations to determine whether OhrA and OhrR contribute to virulence in *C. violaceum*.

In summary, in this work we provide evidence that: (i) OhrA/OhrR is the major pathway involved in the reduction of organic hydroperoxides in *C. violaceum*; (ii) Cys21 is the reactive cysteine, whereas Cys126 is the resolving cysteine of OhrR; (iii) oxidation of OhrR by organic hydroperoxides causes its inactivation; and (iv) OhrR intermolecular disulfide is specifically reduced by thioredoxin, regenerating this thiol-based regulator.

## Materials and Methods

### Bacterial strains and growth conditions

All bacterial strains and plasmids used in this study are listed in Table 1. *Chromobacterium violaceum* type strain ATCC 12472 was used as the wild type strain. *C. violaceum* was grown aerobically at 30°C in Luria-Bertani (LB) medium. Plasmids were introduced into *C. violaceum* by conjugation with *Escherichia coli* strain S17-1. *E. coli* was grown at 37°C in LB medium. When necessary, antibiotics were used at the following concentrations: ampicillin (100 µg ml^−1^), kanamycin (50 µg ml^−1^) and tetracycline (10 µg ml^−1^). The sequences of all DNA oligonucleotide primers are listed in Table 2.

**Table 1:** Bacterial strains and plasmids used in this work.

Strain or plasmid	Description	Reference or source
***E. coli***		
DH5α	*E. coli* strain for cloning purposes	[56]
S17-1	*E. coli* strain for plasmid mobilization	[57]
BL21 (DE3)	*E. coli* strain for protein expression	Novagen
AD494(DE3)	*E. coli* strain for protein expression	Novagen
***C. violaceum***		
ATCC 12472	Wild type strain with sequenced genome	[39]
JF0209	ATCC 12472 *ΔohrA*, deletion mutant	This study
JF0210	ATCC 12472 *ΔohrR*, deletion mutant	This study
**Plasmids**		
pGEM-T Easy	Cloning vector, Amp^r^	Promega
pProEX HTa	Overexpression vector, Amp^r^	Invitrogen
pNPTS138	Suicide vector, containing *oriT sacB*, Kan^r^	D. Alley
pMR20	Broad-host-range low copy vector, Tet^r^	[58]
pET15b-*lpd*	*lpd* coding region cloned in pET15b	[23]
pET15b-*lpdA*	*lpdA* coding region cloned in pET15b	[23]
pET15b-*grxC*	*grxC* coding region cloned in pET15b	A.Y.S. Sakugawa
pPROEX-*ohrR*	*ohrR* coding region cloned in pProEX HTa	This study
pPROEX-C7S	*ohrR*_C7S_ coding region in pProEX HTa	This study
pPROEX-C21S	*ohrR*_C21S_ coding region in pProEX HTa	This study
pPROEX-C126S	*ohrR*_C126S_ coding region in pProEX HTa	This study
pPROEX-C143S	*ohrR*_C143S_ coding region in pProEX HTa	This study
pMR-*ohrA*	pMR20 containing the entire *ohrA* gene	This study
pMR-*ohrR*	pMR20 containing the entire *ohrR* gene	This study
pMR-C7S	pMR20 containing the entire *ohrR*_C7S_	This study
pMR-C21S	pMR20 containing the entire *ohrR*_C21S_	This study
pMR-C126S	pMR20 containing the entire *ohrR*_C126S_	This study
pMR-C143S	pMR20 containing the entire *ohrR*_C143S_	This study
pNPTΔ*ohrA*	*ohrA* flanking regions cloned in pNPTS138	This study
pNPTΔ*ohrR*	*ohrR* flanking regions cloned in pNPTS138	This study

**Table 2:** Primers used in this study.

Primers	Sequence (5′ to 3′)^a^
CV0209-del1	TGGGCCCCCTCCGTCTGCAACGATTCGC
CV0209-del2	TGGATCCCAGAGGGTTCATCTGGCTCTC
CV0209-del3	TGGATCCGCGTAATCGGGCGTACGAATG
CV0209-del4	TGAATTCCTGTTCGAATGGACGGTGTGG
CV0210-del1	TGGGCCCCTTTCAACGATTGCGGGCCGGA
CV0210-del2	TGGATCCCGGCGGGCACGTGTTTTCTTGT
CV0210-del3	TGGATCCTGAACCCGTCAGGGTTTTGATAG
CV0210-del4	TGAATTCGACGAGTTCGTGATCCTGATG
CV0210Exp-Fw	TGAATTCATGGAACAAGAAAACACGTGCCCG
CV0210Exp-Rv	TAAGCTTTCATTGCAGCAGCCGGTCGCGC
pCV0209-Fw	TGGATCCTGCCGGAGCGGAATGCAACT
pCV0209-Rv	TAAGCTTGCGGTGGCTTCGGCGGTATAC
pCV0210-Fw	TGGATCCCTCGCTGGCCGATCTGGGAC
pCV0210-Rv	TAAGCTTGGCGGAATACAGCGCGAAGC
pCV2493-Fw	TGAATTCAATCCGTGACGCCGCACAGG
pCV2493-Rv	TAAGCTTACGGTGGCTTGGGTCCGGTA
CV0209NB-Fw	TGCAGGTGAAGTTGAGCACCCC
CV0209NB-Rv	CCGATGAAGCAGGCGGAATAGCC
CV0210NB-Fw	GGCTGTACCTCGACTCGGCGAC
CV0210NB-Rv	CTCGGTCAGCCGCACAATCACC
CV2493NB-Fw	GTTACGCCGCCTGCTTTCTCGG
CV2493NB-Rv	CGCTGGGAAGGGTGCCGATTC
CV0209CP-Rv	TAAGCTTAATCGTGGATTAGGCCTCGCCG
OhrRC7S-Fw	GGAACAAGAAAACACGAGCCCGCCGCAGGACGCGC
OhrRC7S-Rv	GCGCGTCCTGCGGCGGGCTCGTGTTTTCTTGTTCC
OhrRC21S-Fw	GGCTGGACCAGCAACTCAGCTTCGCGCTGTATTCC
OhrRC21S-Rv	GGAATACAGCGCGAAGCTGAGTTGCTGGTCCAGCC
OhrRC126S-Fw	CCGCTGCAATTGTTGAGCCGGAGCGGAATGCAACTG
OhrRC126S-Rv	CAGTTGCATTCCGCTCCGGCTCAACAATTGCAGCGG
OhrRC143S-Fw	GATTGAGGCAGTCGCTGAGCGATCTGCGCGACCGG
OhrRC143S-Rv	CCGGTCGCGCAGATCGCTCAGCGACTGCCTCAATC

a Underlined letters indicate the restriction enzyme recognition sites, used for cloning purposes.

### Synthesis of linoleic acid and oleic acid hydroperoxides

Linoleic acid hydroperoxide (LaOOH) and oleic acid hydroperoxide (OaOOH) were chemically synthesized by photoxidation of their respective fatty acids with singlet oxygen [59]. LaOOH and OaOOH were separated by silica gel column chromatography, and LaOOH was quantified by its UV absorbance (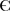
_254 nm_ = 25×10^3^ M^−1^ cm^−1^), considering that 60% of the hydroperoxides contain conjugated diene. The OaOOH concentration was determined by iodometry.

### Construction and complementation of *ohrA* and *ohrR* mutant strains

*C. violaceum* deletion mutant strains were constructed by allelic exchange using a two-step selection procedure and the suicide vector pNPTS138, as previously described [60]. Briefly, two fragments containing regions upstream and downstream of the *ohrA* and *ohrR* genes were PCR-amplified, cloned into the vector pGEM-T Easy, sequenced and subcloned sequentially into the vector pNPTS138. The primers used for *ohrA* were CV0209-del1/CV0209-del2 and CV0209-del3/CV0209-del4, and those used for *ohrR* were CV0210-del1/CV0210-del2 and CV0210-del3/CV0210-del4. The resulting target vectors, pNPTΔ*ohrA* and pNPTΔ*ohrR*, were transferred to *C. violaceum* ATCC 12472 by conjugation. The transconjugants were selected on LB agar medium containing ampicillin (100 µg ml^−1^) and kanamycin (50 µg ml^−1^). The integrants were further screened by growth on LB agar plates containing 10% sucrose. The mutant strains Δ*ohrA* and Δ*ohrR* were confirmed by PCR and designated JF0209 and JF0210, respectively. For complementation of the Δ*ohrA* mutant, a DNA fragment containing the entire *ohrA* gene (680 bp) was PCR-amplified using the primers pCV0209-Fw/CV0209CP-Rv, cloned into pGEM-T Easy, and then moved into pMR20, generating the plasmid pMR-*ohrA*. Similarly, for complementation of the Δ*ohrR* mutant, a DNA fragment containing the full *ohrR* gene (726 bp) was PCR-amplified using the primers pCV0210-Fw/pC0209-Rv, cloned into pGEM-T Easy, and subcloned into pMR20 using *BamH*I and *Hind*III, resulting in the plasmid pMR-*ohrR*. These constructs were introduced into mutant strains by conjugation, and transconjugants were selected in LB agar medium containing ampicillin (100 µg ml^−1^) and tetracycline (10 µg ml^−1^).

### Growth curves

Bacterial cells were grown overnight in LB medium and then diluted to an optical density at 600 nm (OD_600_) of 0.01 in 50 ml of LB medium. Cultures grown until midlog phase during 4 h (OD_600_ ∼0.4) were divided and either untreated or treated with 140 µM *tert*-butyl hydroperoxide, 60 µM cumene hydroperoxide or 75 µM H_2_O_2_. Aliquots of either untreated or treated cultures were collected at several time intervals, and the growth of strains was evaluated by measurement of optical density at 600 nm.

### Site-directed mutagenesis of OhrR

The four cysteine residues of OhrR were replaced with serine by using the QuikChange II site-directed mutagenesis kit (Stratagene). The plasmid pPROEX-OhrR was used as a PCR template with the following complementary mutagenic primers: OhrRC7S-Fw/OhrRC7S-Rv, OhrRC21S-Fw/OhrRC21S-Rv, OhrRC126S-Fw/OhrRC126S-Rv and OhrRC143S-Fw/OhrRC143S-Rv, resulting in four expression vectors with the corresponding mutated *ohrR* gene. To produce these four OhrR mutant proteins *in vivo*, the same complementary mutagenic primers were used, but the plasmid pGEM-OhrR was used as a PCR template. The obtained mutated OhrR genes were then cloned into pMR20 using *BamH*I and *Hind*III, resulting in pMR-C7S, pMR-C21S, pMR-C126S and pMR-C143S, all of which were introduced into the *ohrR* mutant strain by conjugation. All mutations, generating single amino acid substitutions, were confirmed by DNA sequencing.

### Expression and purification of OhrR wild type and mutant proteins

The coding region of the *C. violaceum ohrR* gene (CV0210) was PCR amplified using primers CV0210Exp-Fw/CV0210Exp-Rv, and the 456-bp *EcoR*I/*Hind*III fragment was cloned into the vector pProEX HTa (Gibco BRL) to express an OhrR containing an N-terminal histidine tag. *E. coli* DH5α was transformed with pProEX HTa vectors containing cloned genes encoding the wild type, C7S, C21S, C126S and C143S OhrR by electroporation. Recombinant proteins were induced with 1 mM isopropyl-D-thiogalactopyranoside (IPTG) from midlog *E. coli* cultures grown for 2 h at 37°C in LB media. The harvested cells were resuspended in purification buffer (50 mM sodium phosphate (pH 7.4), 300 mM NaCl, 50 mM imidazole) and lysed by sonication. The His-tagged proteins were then purified from the soluble fraction by NTA-resin affinity chromatography according to the manufacturer's recommendations (Qiagen). The degree of purification was verified by 14% sodium dodecyl sulfate-polyacrylamide gel electrophoresis (SDS-PAGE) under reducing conditions (100 mM DTT).

### Hydroperoxide degradation assay

Degradation of peroxides was measured using previously described methods [14], [55], which were modified from the colorimetric ferrous oxidation-xylenol orange (FOX) assay [61]. Cultures grown in LB medium to an OD at 600 nm of 0.7 were divided and either treated with 300 µM *tert*-butyl hydroperoxide and 200 µM cumene hydroperoxide or 500 µM H_2_O_2_. At 5-min intervals, 1 ml of the culture was collected, centrifuged and 100 µl of the cleared supernatant was then added to 400 µl of 25 mM sulfuric acid. Once all the samples were prepared, 500 µl of freshly prepared iron-xylenol solution (100 µM xylenol orange (Sigma), 2.5 mM ferrous sulfate heptahydrate (Sigma), 3.6 mM butylated hydroxytoluene (Sigma), and 25 mM sulfuric acid, prepared in a methanol∶water (9∶1) solution) were then added to the mixture. After 30 min of incubation at 37°C, the absorbance at 560 nm was determined. As a control, the levels of residual peroxides remaining in the LB medium were also determined.

### RNA isolation and Northern blot analysis

Exponential phase cultures (OD at 600 nm of 0.7) grown in LB medium were divided into flasks, and oxidants or other chemicals were added as described in the legends of the respective figures. After 15 min, untreated or treated cells were centrifuged at 10,000 *g* (4°C), and total RNA was isolated using the TRIzol reagent (Invitrogen) following the manufacturer's protocol. For Northern blot analyses, 15 µg of each RNA sample was subjected to electrophoresis in a 1.5% formaldehyde gel and then transferred to a Hybond-N^+^ membrane (Amersham Biosciences) by passive transfer capillary in 10× SSC (1× SSC is 0.15 M NaCl and 0.015 M sodium citrate) for 15 h. The probes, containing the internal regions of *ohrA* (103 bp), *ohrR* (119 bp) and *ohrB* (114 bp), were PCR-amplified using the primers CV0209NB-Fw/CV0209NB-Rv, CV0210NB-Fw/CV0210NB-Rv and CV2493NB-Fw/CV2493NB-Rv, respectively. The probes were end labeled with T4 polynucleotide kinase (Fermentas) using [γ^32^P]-ATP. The membranes were pre-hybridized in 15 ml of ULTRAhyb (Ambion) at 42°C for 30 min and incubated with the labeled probes at 42°C for 3 h. The membranes were washed twice at 42°C for 5 min in 2× SSC plus 0.1% SDS and twice at 42°C for 15 min in 0.1× SSC plus 0.1% SDS. The results were detected by autoradiography.

### Primer extension assays

Total RNA samples, isolated as described above, were subjected to primer extension assays to determine transcription start sites using the primer pCV0209-Rv, specific to *ohrA*, and the primer pCV0210-Rv, specific to *ohrR*. Approximately one picomol of primers, end labeled with [γ^32^P]-ATP using T4 polynucleotide kinase (Fermentas), was hybridized to 10 µg of total RNA for 2 h (58°C). The primers were extended with ImProm-II reverse transcriptase for 1 h (47°C), according to the manufacturer's recommendations (Promega). Extension products were phenol extracted, ethanol precipitated and run on a polyacrylamide sequencing gel alongside a sequencing ladder generated using the fmol DNA cycle sequencing system (Promega) and the promoter fragments as templates.

### Electrophoretic mobility shift assays (EMSAs)

DNA fragments covering the promoter regions of the *ohrA* (211 bp), *ohrR* (221 bp) and *ohrB* (253 bp) genes were obtained by PCR amplification using the primers pCV0209-Fw/pCV0209-Rv, pCV0210-Fw/pCV0210-Rv and pCV2493-Fw/pCV2493-Rv, respectively. DNA probes were end labeled with [γ^32^P]-ATP using T4 polynucleotide kinase (Fermentas), and the unincorporated nucleotides were removed using a Wizard SV PCR Clean-up system (Promega). DNA-binding reactions were performed in a mixture containing 20 mM Tris-HCl (pH 7.4), 50 mM KCl, 1 mM dithiothreitol (DTT), 1 mM EDTA, 5% glycerol, 0.05 mg/ml bovine serum albumin (BSA), 0.1 mg/ml competitor salmon sperm DNA, labeled DNA probes, and various amounts of OhrR proteins. The samples were incubated at 25°C for 25 min and loaded on a 5% polyacrylamide gel in 0.5× Tris borate-EDTA buffer. The gel was dried and analyzed by autoradiography.

### Investigation of biological reducing systems for OhrR

The abilities of different reductants to reduce OhrR were tested using EMSA with the *ohrA* promoter region probe. Samples of air-oxidized OhrR (5 µM) were incubated in 10 µl of binding buffer (without DTT in this case) for 30 min at 30°C with the following treatments: 10, 20 and 40 µM *E. coli* thioredoxin (TrxA), 1 and 5 µM *E. coli* thioredoxin reductase (TrxB) and 0.5 mM NADPH for the thioredoxin system; 1, 10 and 20 µM *X. fastidiosa* glutaredoxin 3 (GrxC), 10 mM glutathione (GSH), 10 µg/ml *Saccharomyces cerevisiae* glutathione reductase (GR) and 0.5 mM NADPH for the glutathione/glutaredoxin system; 1, 10 and 50 µM *X. fastidiosa* LpdA and 0.5 mM NADH; 10 µM lipoamide, 1, 5 and 10 µM *X. fastidiosa* dihydrolipoamide dehydrogenase (Lpd) and 0.5 mM NADH for the lipoamide system; and 1 and 10 mM DTT as positive controls. After treatments, all samples were diluted 20 times to obtain OhrR at 250 nM prior to analysis by EMSA. The disulfide reductase activities of the reducing systems were determined using the 5,5′-dithiobis-(2-nitrobenzoic acid) (DTNB) assay for thioredoxin and the lipoamide systems or the 2-hydroxyethyl disulfide (HED) assay for the glutaredoxin system, as previously described [23], [62]. Lpd and LpdA recombinant proteins (from *Xylella fastidiosa*) were expressed and purified as previously described [23]. TrxA (catalog no. T0910) and TrxB (catalog no. T7915) from *E. coli*, glutathione reductase from baker's yeast (catalog no. G3664), NADPH, NADH, GSH and lipoamide were purchased from Sigma.

### DNase I footprinting

The same DNA fragments used for the EMSA assays, containing the *ohrA* and *ohrR* promoters, were PCR-amplified using only the reverse primers end-labeled. Binding reactions were performed as described in the EMSA assay without EDTA addition. DNase I treatment and gel electrophoresis were performed as previously described [60]. A sequence ladder was generated using the fmol DNA cycle sequencing system (Promega) and the promoter fragments as templates.

### Analysis of disulfide bond formation in OhrR by nonreducing SDS-PAGE

Wild type and C7S, C21S, C126S and C143S OhrR variants were reduced by incubating the purified proteins (28 µM) in 100 mM of DTT for 2 h and desalted using PD-10 gel filtration columns (GE Healthcare). To obtain these proteins in the oxidized state, the reduced samples of OhrR (10 µM) were treated with 100 µM cumene hydroperoxide for 30 min. To prevent nonspecific disulfide bond formation, all samples were treated with 100 mM *N*-ethylmaleimide (NEM) for 2 h prior to analysis by 14% nonreducing SDS-PAGE.

## Supporting Information

Figure S1 **EMSA assay of OhrR with** ***ohrB*** **.** (**A**) Labeled probe containing the promoter region of *ohrB* was incubated with the indicated concentrations of purified OhrR. (**B**) Negative control: a probe of the *ohrR* coding region incubated with increasing concentrations of purified OhrR protein (0 to 5000 nM).
(TIF)

Figure S2 **Disulfide reductase activity of different reductive systems.** The activities of the thioredoxin system (**A**), LpdA (**B**) and Lpd (**C**) (lipoamide systems) were determined by the DTNB assay. The reaction mixtures contain 50 mM sodium phosphate (pH 7.4), 1 mM diethylenetriamine pentaacetic acid (DTPA), 0.5 mM DTNB and 0.2 mM NAD(P)H. (**A**) Thioredoxin system: 0.1 µM TrxB (diamond), 5 µM TrxA (square) or 0.1 µM TrxB plus 5 µM TrxA (triangle). (**B**) Lipoylated enzyme LpdA (5 µM) was added (triangle) or omitted as a control (square). (**C**) Lipoamide system: 40 µM lipoamide (open diamond), 5 µM Lpd (closed diamond) or 40 µM lipoamide plus 5 µM Lpd (triangle). (**D**) The disulfide reductase activity of the glutaredoxin system was determined by the HED assay monitoring NADPH oxidation. Mixture reactions contain 20 mM Tris-Cl (pH 7.4), 0,1 mg/ml BSA, 0,7 mM HED, 0,2 mM NADPH, 1 mM glutathione, 10 µg/ml glutathione reductase, and 5 µM glutaredoxin 3 (GrxC) (triangle). As a control, GrxC was omitted from the assay mixture (diamond).
(TIF)
