# Supplementary figures and images for: Analysis of the Organic Hydroperoxide Response of Chromobacterium violaceum Reveals That OhrR Is a Cys-Based Redox Sensor Regulated by Thioredoxin
Source: PLoS One. 2012 Oct 11;7(10):e47090. doi: 10.1371/journal.pone.0047090 (PMC3469484; doi:10.1371/journal.pone.0047090)

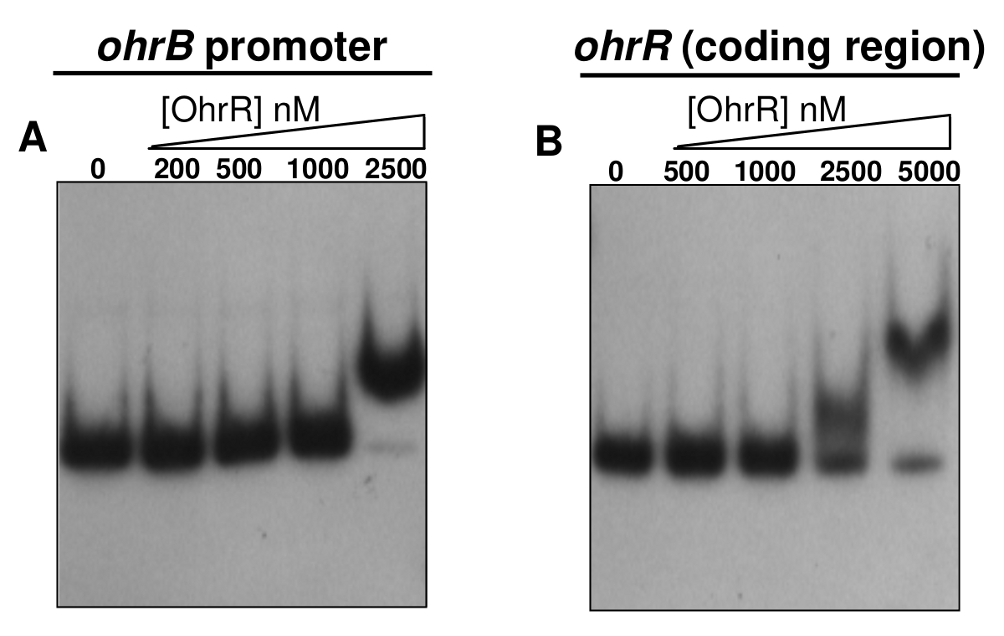

Supplement: Figure S1 — EMSA assay of OhrR with ohrB . (A) Labeled probe containing the promoter region of ohrB was incubated with the indicated concentrations of purified OhrR. (B) Negative control: a probe of the ohrR coding region incubated with increasing concentrations of purified OhrR protein (0 to 5000 nM). (TIF) [file pone.0047090.s001.tif]

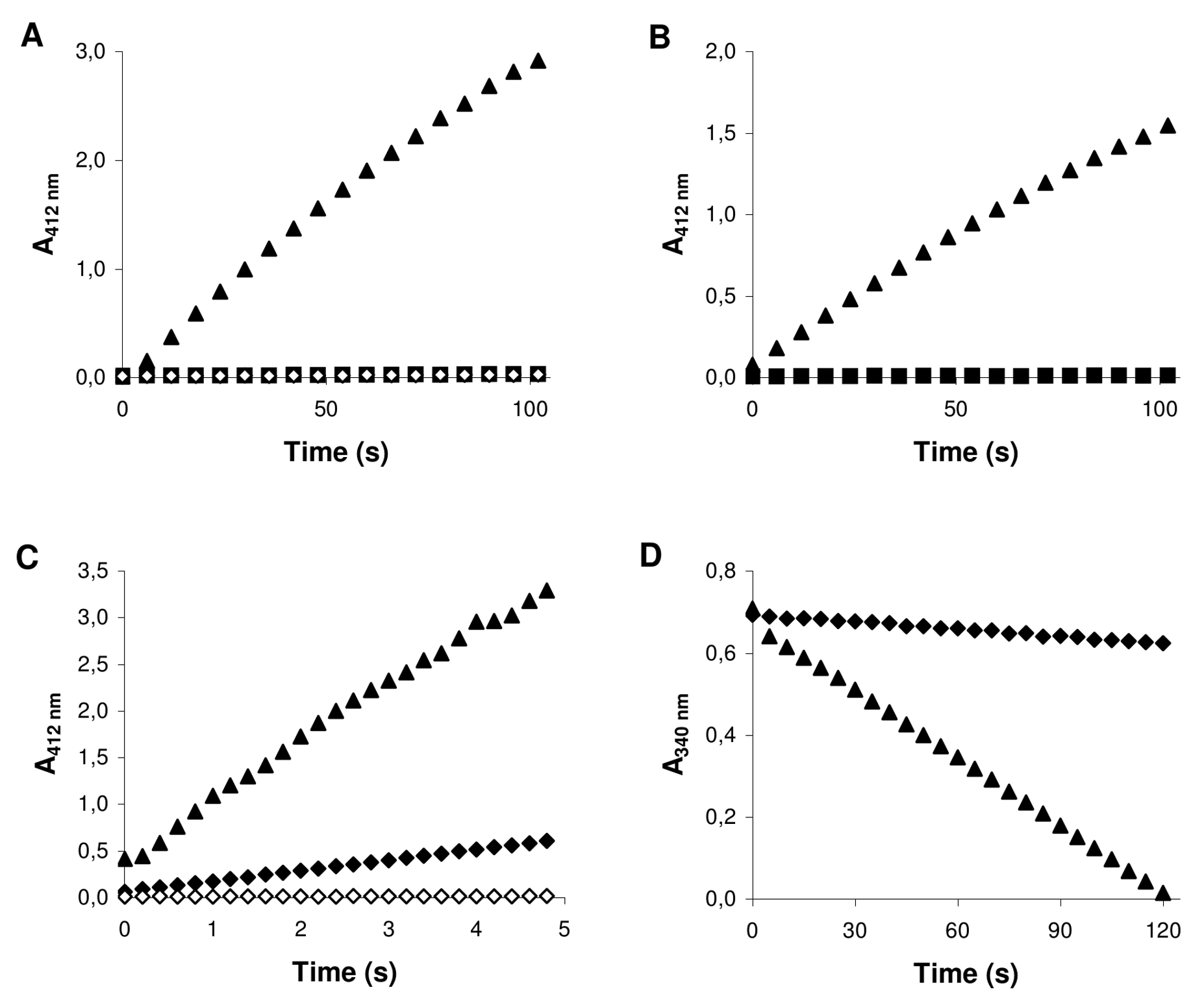

Supplement: Figure S2 — Disulfide reductase activity of different reductive systems. The activities of the thioredoxin system (A), LpdA (B) and Lpd (C) (lipoamide systems) were determined by the DTNB assay. The reaction mixtures contain 50 mM sodium phosphate (pH 7.4), 1 mM diethylenetriamine pentaacetic acid (DTPA), 0.5 mM DTNB and 0.2 mM NAD(P)H. (A) Thioredoxin system: 0.1 µM TrxB (diamond), 5 µM TrxA (square) or 0.1 µM TrxB plus 5 µM TrxA (triangle). (B) Lipoylated enzyme LpdA (5 µM) was added (triangle) or omitted as a control (square). (C) Lipoamide system: 40 µM lipoamide (open diamond), 5 µM Lpd (closed diamond) or 40 µM lipoamide plus 5 µM Lpd (triangle). (D) The disulfide reductase activity of the glutaredoxin system was determined by the HED assay monitoring NADPH oxidation. Mixture reactions contain 20 mM Tris-Cl (pH 7.4), 0,1 mg/ml BSA, 0,7 mM HED, 0,2 mM NADPH, 1 mM glutathione, 10 µg/ml glutathione reductase, and 5 µM glutaredoxin 3 (GrxC) (triangle). As a control, GrxC was omitted from the assay mixture (diamond). (TIF) [file pone.0047090.s002.tif]
